# Disruption of the mouse *Slc39a14* gene encoding zinc transporter ZIP14 is associated with decreased bone mass, likely caused by enhanced bone resorption

**DOI:** 10.1002/2211-5463.12399

**Published:** 2018-02-26

**Authors:** Sun Sasaki, Manami Tsukamoto, Masaki Saito, Shintaro Hojyo, Toshiyuki Fukada, Masamichi Takami, Tatsuya Furuichi

**Affiliations:** ^1^ Laboratory of Laboratory Animal Science and Medicine Co‐Department of Veterinary Medicine Faculty of Agriculture Iwate University Morioka Japan; ^2^ Osteoimmunology Deutsches Rheuma‐Forschungszentrum Berlin Germany; ^3^ Faculty of Pharmaceutical Sciences Tokushima Bunri University Tokushima Japan; ^4^ Department of Pathology School of Dentistry Showa University Tokyo Japan; ^5^ RIKEN Center for Integrative Medical Sciences Yokohama Japan; ^6^ Department of Pharmacology School of Dentistry Showa University Tokyo Japan; ^7^ Department of Basic Veterinary Science United Graduate School of Veterinary Science Gifu University Gifu Japan

**Keywords:** bone homeostasis, knockout mouse, osteoclast, zinc transporter, ZIP14

## Abstract

Osteoclasts are bone‐resorbing cells that play an essential role in maintaining bone homeostasis. Zinc (Zn) has been reported to inhibit osteoclast‐mediated bone resorption, but the mechanism of this action has not been clarified. Zn homeostasis is tightly controlled by the coordinated actions of many Zn transporters. The Zn transporter ZIP14/Slc39a14 is involved in various physiological functions; hence, *Zip14*‐knockout (KO) mice exhibit multiple phenotypes. In this study, we thoroughly investigated the bone phenotypes of *Zip14*‐KO mice, demonstrating that the KO mice exhibited osteopenia in both trabecular and cortical bones. In *Zip14*‐KO mice, bone resorption was increased, whereas the bone formation rate was unchanged. *Zip14* mRNA was expressed in normal osteoclasts both *in vivo* and *in vitro*, but receptor activator of NF‐κB ligand (RANKL)‐induced osteoclastogenesis was not impaired in bone marrow‐derived macrophages prepared from *Zip14*‐KO mice. These results suggest that ZIP14 regulates bone homeostasis by inhibiting bore resorption and that in *Zip14*‐KO mice, bone resorption is increased due to the elimination of this inhibitory regulation. Further studies are necessary to conclude whether the enhancement of bone resorption in *Zip14*‐KO mice is due to a cell‐autonomous or a non‐cell‐autonomous osteoclast defect.

**Abbreviations**
BMM: bone marrow‐derived macrophage
IL: interleukin
KO: knockout
LPS: lipopolysaccharide
M‐CSF: macrophage colony‐stimulating factor
NFATc1: nuclear factor‐activated T cells, cytoplasmic, calcineurin‐dependent 1
RANKL: receptor activator of NF‐κB ligand
sIL‐6R: soluble IL‐6 receptor
TGF: transforming growth factor
TRAP: tartrate‐resistant acid phosphatase

Zinc (Zn) is an essential trace element for various biological activities 1 as it is needed for more than 300 enzymes and it serves as a structural component of at least 3000 proteins in the body. Recently, the Zn ion (Zn^2+^) has been reported to act as a second messenger that regulates intracellular signal transduction in various cell types 2. The Zn concentration is relatively high in bone and cartilage 3, and Zn deficiency delays skeletal growth and decreases bone mass 4, 5. These findings indicate that Zn homeostasis is important for skeletal development and maintenance.

During adulthood, bone mass is maintained by the coupled activities of osteoblasts and osteoclasts 6, 7. In this physiological process, old bone is resorbed by osteoclasts and then replaced by new bone formed by osteoblasts. An imbalance between these processes leads to bone metabolic diseases, such as osteoporosis and occasionally osteopetrosis. Several *in vitro* studies illustrated that Zn stimulates osteoblast‐mediated bone formation and inhibits osteoclast‐mediated bone resorption, thereby positively regulating bone mass 8, 9. However, the details of these Zn‐mediated regulatory mechanisms have not yet been clarified.

Zn homeostasis is tightly controlled by two major families of Zn transporters: SLC39s/ZIPs and SLC30s/ZnTs 10, 11. ZIP transporters promote Zn influx from extracellular fluid or intracellular vesicles into the cytoplasm, whereas ZnT transporters promote Zn efflux from cells or influx into intracellular vesicles from the cytosol. At least 14 ZIP and 10 ZnT transporters have been identified in mammals. Among them, two Golgi‐localized transporters, namely ZIP13 and ZnT5, have been reported to positively regulate osteoblast differentiation and/or function 12, 13, 14. ZIP13 is expressed in osteoblasts, and *Zip13*‐knockout (KO) mice display decreased bone mass and bone formation rate. Furthermore, primary osteoblasts isolated from *Zip13*‐KO mice exhibited impaired expression of the osteoblast marker genes. *Znt5*‐KO mice also display decreased bone mass and bone formation rate, and alkaline phosphatase and mineralization activities are diminished in primary *Znt5*‐KO osteoblasts. The plasma membrane Zn transporter, ZIP1, is reported to be expressed in osteoclasts 15. Adenoviral overexpression of ZIP1 in osteoclasts reduces bore resorption activity *in vitro,* suggesting that ZIP1 negatively regulates osteoclast function.

ZIP14/Slc39a14 localizes to cell membranes and promotes Zn influx into cells 16, 17. It has been reported that ZIP14 is expressed ubiquitously and it transports other metals, such as manganese (Mn), iron (Fe), and cadmium (Cd), in addition to Zn 17, 18, 19. In accordance with these findings, *Zip14*‐KO mice have been reported to exhibit multiple phenotypes, including dwarfism, osteopenia, altered glucose homeostasis, low‐grade chronic inflammation, and increased body fat 20, 21, 22, 23. In skeletal tissues, ZIP14 is expressed in the growth plate chondrocytes, in which it regulates differentiation 14, 20. In this study, we comprehensively examined the bone phenotypes of *Zip14*‐KO mice. We focused on the ZIP14 function as a Zn transporter because Zn is more important for skeletal development and homeostasis than other metals transported by ZIP14. *Zip14*‐KO mice exhibited an osteopenia phenotype accompanied by enhanced bone resorption, and ZIP14 was expressed in normal osteoclasts. These findings strongly suggest that ZIP14 regulates bone homeostasis by affecting osteoclast‐mediated bore resorption.

## Materials and methods

### Experimental animals

*Zip14*‐KO mice were generated as described previously and maintained on a C57BL/6 background 20. Mice were housed in a temperature‐controlled room with a 12‐h/12‐h light/dark cycle. Mice had free access to water, and they were fed standard mouse laboratory chow. Genotyping of mice was performed at 4–5 weeks of age by PCR as described previously 20. All of the animal experiments were performed according to a protocol approved by Iwate University's Committee on Animal Research and Ethics (Approval Numbers: 201214 and 201512).

### X‐ray and pQCT analyses

Femurs were dissected from sacrificed mice and fixed with ethanol. Radiographs were obtained using a TRS‐1005 soft X‐ray apparatus (Sofron, Tokyo, Japan). Femoral cortical bone quality was measured by pQCT analysis using an XCT Research SA+ computed tomography system (Stratec Medizintechnik GmbH, Pforzheim, Germany).

### Histological analyses

To assess dynamic histomorphometric indices, 6‐week‐old mice were injected twice with calcein (15 mg·kg^−1^, i.p.) at 1 and 4 days before sacrifice. Tibiae were fixed with ethanol, and the undecalcified bones were embedded in glycol methacrylate. Sections (3 μm thick) were cut longitudinally in the proximal region of the tibia and stained with toluidine blue O. Histomorphometry was performed using a semiautomatic image analyzing system (Osteoplan II; Carl Zeiss, Thornwood, NY, USA) linked to a light microscope. The histomorphometric measurements were performed at 400 times using a minimum of 17–20 optical fields in the secondary spongiosa area from the growth plate–metaphyseal junction. Nomenclature, symbols, and units were used as recommended by the Nomenclature Committee of the American Society for Bone and Mineral Research. For *in situ* hybridization, paraffin sections (4–6 μm thick) were prepared from the tibiae of mice at 4 weeks of age. The digoxigenin‐labeled RNA probe for *Zip14* was used in line with a method described by GENOSTAFF CO., LTD (Tokyo, Japan). The sections were counterstained with Kernechtrot stain solution. The probe sequences and hybridization conditions are available upon request.

### *In vitro* assay for osteoclastogenesis

To prepare bone marrow‐derived macrophages (BMMs), bone marrow cells were collected from the tibiae and femurs of mice at 6–10 weeks of age and cultured for 16 h in αMEM containing 10% FBS, 50 ng·mL^−1^ macrophage colony‐stimulating factor (M‐CSF), and 1 ng·mL^−1^ transforming growth factor (TGF)‐β1 in cell culture dishes. M‐CSF (Leukoprol) and TGF‐β1 were purchased from Kyowa Hakko Kogyo (Tokyo, Japan) and R&D Systems (Minneapolis, USA), respectively. Supernatants were transferred to Petri dishes and cultured for 72 h. Adherent cells were collected and used as BMMs. For osteoclast formation assays, BMMs (4 × 10^4^ cells per well) cells were cultured for 3–4 days in 48‐well culture plates in the presence of 50 ng·mL^−1^ M‐CSF, 1 ng·mL^−1^ TGF‐β1, and 20 or 100 ng·mL^−1^ receptor activator of NF‐κB ligand (RANKL; R&D Systems). After cultivation, tartrate‐resistant acid phosphatase (TRAP) staining and TRAP activity assays were performed as described previously 24. To evaluate bone‐resorbing activity, areas of pits eroded by osteoclasts were measured. BMMs (1.5 × 10^4^ cells per well) were seeded onto Corning OsteoAssay Surface 96‐well plates (Corning Incorporated, Corning, NY, USA) in the presence of 50 ng·mL^−1^ M‐CSF, 1 ng·mL^−1^ TGF‐β1, and 100 ng·mL^−1^ RANKL for 7 days. The culture medium was changed every 3 days. Cells were removed using 5% sodium hypochlorite, followed by washing with distilled water and air‐drying. Resorption pits were visualized under a scanning electron microscope, and the resorption area was quantified using imagej software (National Institutes of Health, Bethesda, MD, USA).

### RT‐PCR

Total RNA was extracted from BMMs at the indicated time points after 100 ng·mL^−1^ RANKL treatment using ISOGEN (Nippon Gene, Tokyo, Japan). First‐stand cDNA was synthesized using TaqMan Multiscribe Reverse Transcriptase (Applied Biosystems, Foster City, CA, USA) and subjected to amplification using Ex Taq polymerase (TaKaRa, Tokyo, Japan) and the following specific PCR primers: 5′‐CAGAGGCTTTTGGCTTCAAC‐3′ and 5′‐CAGACACAGTGAAGGAGGCA‐3′ for *Zip14*; 5′‐ACTCCTGGGATCAACGTGAC‐3′ and 5′‐GATAGCACATAGGGGGCAGA‐3′ for *Oscar*; 5′‐TCTCTGCCCATAACCTGGAG‐3′ and 5′‐TACAACTTTCATCCTGGCCC‐3′ for *Ctsk*; and 5′‐AACTGGGACGACATGGAGAA‐3′ and 5′‐GGGGTGTTGAAGGTCTAAA‐3′ for *Gapdh*. The PCR conditions are available upon request.

### Measurement of serum TRAP levels

Tartrate‐resistant acid phosphatase is used as a serum marker for bone resorption. Serum TRAP levels were determined using a mouse TRAP™ Assay kit (Immunodiagnostic Systems, Boldon, UK).

### Statistical analysis

Student's *t*‐test was used to determine the significance of differences between control (Ctrl) and *Zip14*‐KO mice. Significance was defined as *P* < 0.05.

## Results

### Both trabecular and cortical bone levels are decreased in *Zip14*‐KO mice

**Figure 1:**
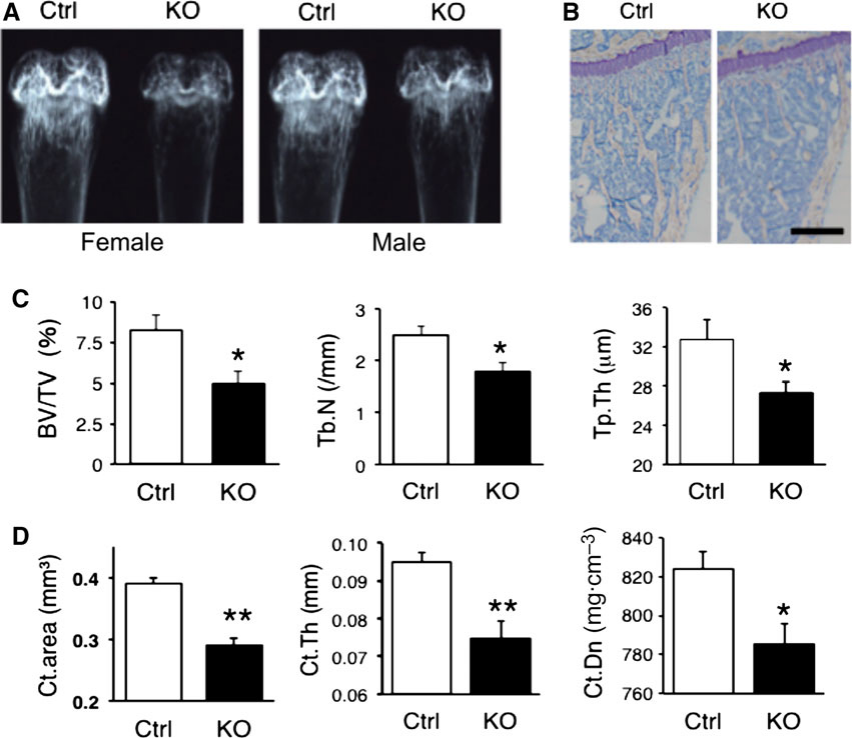
Both trabecular and cortical bone levels are decreased in *Zip14*‐KO mice. (A) X‐ray images of distal femurs from 6‐week‐old female and male mice. (B) Histological images of the proximal tibiae of 6‐week‐old female mice. Scale bar, 500 μm. (C) Trabecular bone parameters of bone histomorphometric analyses using proximal tibia sections from 6‐week‐old mice. Trabecular bone volume per total volume (BV/TV), trabecular number (Tb.N), and trabecular thickness (Tb.Th) were compared between control (Ctrl) and *Zip14*‐KO mice. (D) Cortical bone parameters of pQCT analyses using the diaphyses of the femurs of 6‐week‐old female mice. Cortical area (Ct.area), cortical thickness (Ct.Th), and cortical density (Ct.Dn) were compared between Ctrl and *Zip14*‐KO mice. (C, D) Data are represented as the mean ± S.E. (*n* = 5). *P < 0.05, ** *P* < 0.01 by Student's t‐test.

To examine the role of ZIP14 in bone homeostasis, we compared the bone phenotypes between Ctrl and *Zip14*‐KO mice. Because the phenotypic abnormalities were inherited recessively in *Zip14*‐KO mice and heterozygous *Zip14*‐KO mice were normal 20, 21, 22, 23, the Ctrl group consisted of both wild‐type and heterozygous *Zip14*‐KO mice. In preliminary studies, we performed X‐ray analyses using the distal femurs of both male and female mice at 6 weeks of age. It appeared that *Zip14*‐KO mice were more radiolucent than Ctrl mice, and the increased radiolucency was comparable between male and female *Zip14*‐KO mice (Fig. 1A). This phenotype appeared at 15 months of age (data not shown). The skeleton is composed of two types of bone, namely cortical bone and trabecular bone. Cortical bone has a higher mineral density, thereby protecting the soft tissues and giving the body its shape. Conversely, trabecular bone, also known as cancellous bone, has a lower mineral density, and it is easily resorbed, thereby playing an important role in calcium homeostasis. Histomorphometric analysis revealed that trabecular bone volume, trabecular number, and trabecular thickness were significantly lower in *Zip14*‐KO mice than in Ctrl mice (Fig. 1B,C). pQCT analysis demonstrated that cortical area, cortical thickness, and cortical density were also decreased in *Zip14*‐KO mice (Fig. 1D). In agreement with the previous reports 20, 22, *Zip14*‐KO mice displayed an osteopenia phenotype, and we demonstrated for the first time that osteopenia in *Zip14*‐KO mice appeared in both trabecular and cortical bones.

### Bone resorption activity was increased in *Zip14*‐KO mice

**Figure 2:**
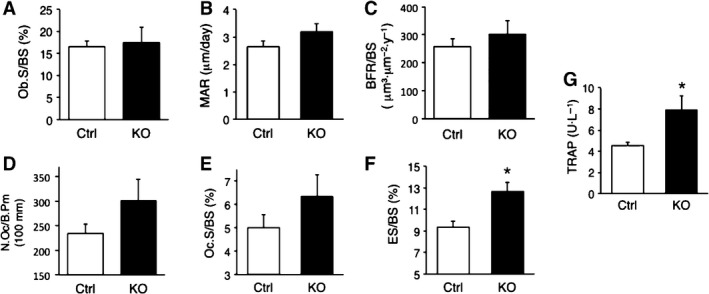
Effect of ZIP14 disruption on bone formation and resorption *in vivo*. (A–F) Bone histomorphometric analyses were performed using proximal tibia sections from 6‐week‐old female mice. Osteoblast surface per bone surface (Ob.S/BS) (A), mineral apposition rate (MAR) (B), bone formation rate per bone surface (BFR/BS) (C), osteoclast number per bone perimeter (N.Oc/B.Pm) (D), osteoclast surface per bone surface (Oc.S/BS) (E), and eroded surface per bone surface (ES/BS) (G) were compared between control (Ctrl) and *Zip14*‐KO mice. Data are represented as the mean ± SE. (*n*=5). (G) Serum TRAP levels measured by ELISA. Data are represented as the mean ± S.E. (Ctrl, *n* = 9; KO, *n* = 7). *P < 0.05 by Student's *t*‐test.

To examine the cause of the osteopenia phenotype in *Zip14*‐KO mice, histomorphometric analysis was performed using calcein‐double‐labeled tibia sections at 6 weeks of age. Concerning three parameters reflecting osteoblast activity (osteoblast surface, mineral apposition rate, and bone formation rate), there were no significant differences between Ctrl and *Zip14*‐KO mice (Fig. 2A–C). Two parameters reflecting osteoclast activity (osteoclast number and osteoclast surface) were increased in *Zip14*‐KO mice compared to the findings in Ctrl mice, albeit without significance (Fig. 2D,E). Conversely, the eroded surface‐to‐bone surface ratio (ES/BS) was significantly increased in *Zip14*‐KO mice (Fig. 2F). ES/BS is defined as the percentage of the bone surface that exhibits signs of past or present resorption, thereby representing osteoclast activity *in vivo*. Further, serum TRAP levels were significantly increased in *Zip14*‐KO mice (Fig. 2G). These results revealed that osteoclast‐mediated bone resorption is increased in *Zip14‐*KO mice.

### *Zip14* mRNA was expressed in osteoclasts both *in vitro* and *in vivo*

**Figure 3:**
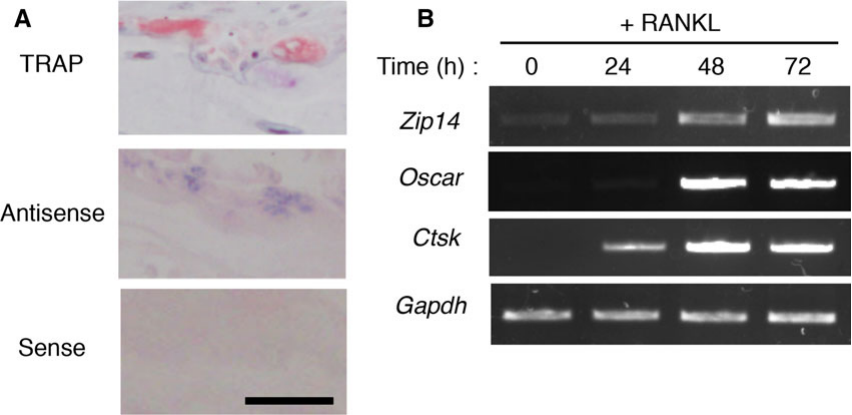
*Zip14* mRNA was expressed in osteoclasts both *in vitro* and *in vivo*. (A) *In situ* hybridization of *Zip14* mRNA using tibia sections from 4‐week‐old control mice. Images of TRAP staining (upper) and *in situ* hybridization using *Zip14* antisense (middle) and sense probes (lower) are shown. The *Zip14* sense probe served as the negative control. Note that positive signals were detected in multinucleated cells on the bone surface using the *Zip14* antisense probe but not the *Zip14* sense probe. Scale bar, 100 μm. (B) RT‐PCR analyses to detect *Zip14* mRNA in BMMs subjected to RANKL‐induced osteoclast differentiation. BMMs were stimulated with M‐CSF (50 ng·mL), TGF‐β1 (1 ng·mL^−1^), and RANKL (100 ng·mL^−1^) for the indicated periods, and *Zip14, Oscar, Ctsk,* and *Gapdh* mRNA expression was examined by RT‐PCR. *Oscar* and *Ctsk* are osteoclast marker genes.

Because bone resorption was increased in *Zip14*‐KO mice, we measured *Zip14* mRNA expression in osteoclasts from Ctrl mice. Osteoclasts are large multinucleated cells with highly TRAP activity that arise from the monocyte/macrophage lineage cells 25. *In situ* hybridization revealed that *Zip14* mRNA was expressed in TRAP‐positive multinucleated cells on the bone surface *in vivo* (Fig. 3A), but it was not expressed in osteoblasts (data not shown). Next, RT‐PCR was performed using cDNA prepared from RANKL‐treated Ctrl BMMs *in vitro* (Fig. 3B). RANKL is a critical cytokine for osteoclast differentiation and activation 26, 27, 28. Two osteoclast maker genes, *Oscar* and *Ctsk*, were abundantly expressed 48 and 96 h after RANKL simulation, indicating that BMMs could differentiate into osteoclasts. *Zip14* mRNA was modestly expressed in untreated BMMs, and its expression gradually increased after RANKL treatment. These results indicate that ZIP14 is expressed in osteoclasts and suggest the involvement of ZIP14 in RANK‐induced osteoclast differentiation.

### Osteoclastogenesis was not impaired in *Zip14*‐KO BMMs *in vitro*

**Figure 4:**
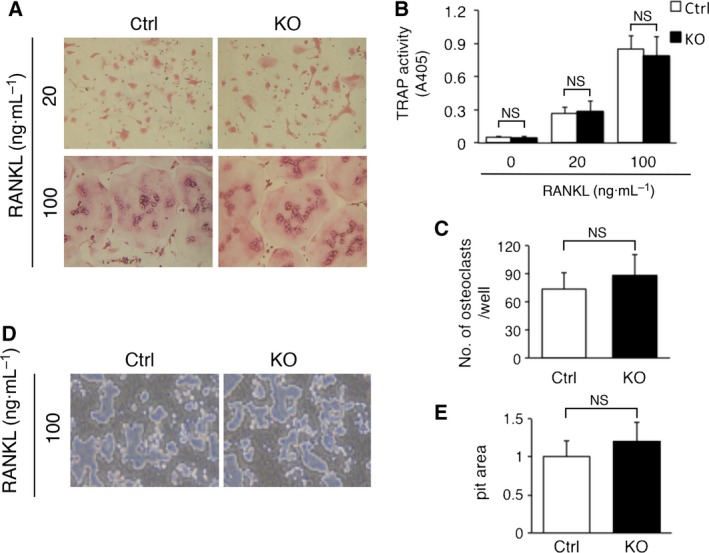
Osteoclastogenesis was not impaired in *Zip14*‐KO BMMs *in vitro*. (A–C) BMMs were cultured with M‐CSF (50 ng·mL^−1^), TGF‐β1 (1 ng·mL^−1^), and RANKL (20 or 100 ng·mL^−1^). Osteoclast formation was evaluated by TRAP staining image (A), TRAP activity (B), and osteoclast number (C, count only when cultured with 100 ng·mL^−1^ RANKL). TRAP‐positive cells with more than three nuclei were identified as osteoclasts. (D,E) BMMs were cultured with M‐CSF (50 ng·mL^−1^), TGF‐β1 (1 ng·mL^−1^), and RANKL (100 ng·mL^−1^) on Corning OsteoAssay Surface 96‐well plates. After cultivation, cells were removed using 5% sodium hypochlorite. Resorption pits were visualized under a scanning electron microscope (D), and the resorption area was quantified using imagej software (E). (B,C,E) Data are presented as mean ± SE (*n* = 5). NS: no statistical difference by Student's *t*‐test.

Finally, we examined the effect of ZIP14 disruption on the osteoclastogenesis in RANKL‐treated BMMs. BMMs prepared from Ctrl and *Zip14*‐KO mice were cultured in the presence of M‐CSF, TGF‐β1, and RANKL. TRAP‐positive cells with more than three nuclei were identified as osteoclasts, and the area of resorption pits formed by osteoclasts on inorganic bone was measured to evaluate bone‐resorbing activity. The images of TRAP‐stained cells were comparable between Ctrl and *Zip14*‐KO mice (Fig. 4A). No significant differences were noted for any parameters, including TRAP activity, osteoclast number, and pit area, between Ctrl and *Zip14*‐KO mice (Fig. 4B,C,E). These results indicate that RANKL‐induced osteoclastogenesis is not impaired in *Zip14*‐KO BMMs *in vitro* and intimate that ZIP14 disruption in osteoclasts is not a major cause of the increased bone resorption in *Zip14*‐KO mice.

## Discussion

*Zip14*‐KO mice exhibited osteopenia with increased bone resorption activity. Although ZIP14 was expressed in normal osteoclasts, osteoclastogenesis was not impaired in *Zip14*‐KO BMMs *in vitro*. It has been reported that ZIP14 is expressed ubiquitously and it transports other metals (Mn, Fe, and Cd), in addition to Zn 17, 18, 19. In agreement with these findings, *Zip14*‐KO mice exhibit multiple phenotypes, indicating that ZIP14 is an ion transporter involved in various physiological responses. However, the main cause of the increased bone resorption in *Zip14*‐KO mice was not precisely determined.

There are two possible explanations for the observation of normal osteoclastogenesis in *Zip14*‐KO BMMs *in vitro*. One possibility is that ZIP14 disruption in osteoclasts increases bore resorption autonomously in *Zip14*‐KO mice; however, this defect cannot be reproduced under *in vitro* culture conditions. Occasionally, cultured cells do not have the same physiological responses as the original cells. It has been reported that the percentage of serum, the source of serum, and presence and absence of serum itself influence the Zn‐dependent responses of many cell types 29. Compensatory upregulation of other Zn transporters may be associated with the normal osteoclastogenesis in *Zip14*‐KO BMMs. If this is true, then ZIP14‐mediated Zn influx into osteoclasts should negatively regulate osteoclastogenesis; that is, the bone resorption is increased in *Zip14*‐KO mice because of the elimination of this negative regulation.

Many *in vitro* studies demonstrated that Zn has inhibitory effects on osteoclast‐mediated bone resorption 8, 9, 30, and the present study suggests that ZIP14‐mediated Zn influx is involved in this effect. Osteoclasts differentiate from the monocyte/macrophage lineage upon stimulation by two essential cytokines, M‐CSF and RANKL 26, 27, 28. Activation of transcription factors such as c‐Fos, NF‐κB, and nuclear factor‐activated T cells, cytoplasmic, calcineurin‐dependent 1 (NFATc1) is required for sufficient osteoclast differentiation. In particular, NFATc1 serves as a master transcriptional regulator of osteoclast differentiation. Oral Zn administration was reported to decrease osteoclastogenesis by inhibiting RANKL expression in Zn‐adequate rats 31. Park *et al*. reported that Zn inhibited osteoclast differentiation *in vitro* by inhibiting Ca^2+^–calcineurin–NFATc1 signaling pathway 32. Phospho‐NFATc1 is dephosphorylated by activated calcineurin, which leads to nuclear translocation of the protein and the induction of NFATc1‐mediated gene transcription. Zn inhibits calmodulin activity by competing with Ca^2+^ for binding to calmodulin, resulting in the inhibition of NFATc1 translocation to the nucleus 33. Yamaguchi *et al*. reported that Zn inhibited osteoclast differentiation *in vitro* by inhibiting NF‐κB activation 30. Adenoviral overexpression of ZIP1 also inhibited NF‐κB activation leading to impaired osteoclast function 15. ZIP14‐mediated Zn influx may influence Ca^2+^–calcineurin–NFATc1 signaling and/or NF‐κB activation during osteoclast differentiation.

Lipopolysaccharide (LPS), a component of the outer membranes of Gram‐negative bacteria, is capable of inducing bone resorption in both *in vitro* and *in vivo* studies 34, 35. LPS induces the production of pro‐inflammatory cytokines such as tumor necrosis factor‐α, interleukin (IL)‐1β, and IL‐6, which can directly stimulate osteoclast differentiation. LPS alone can induce osteoclast differentiation in RAW264.7 macrophage cells but not in BMM culture 36, 37. LPS was reported to strongly induce *ZIP14* mRNA in primary macrophage cells prepared from human blood 38. Therefore, upregulation of ZIP14 expression may be involved in LPS‐induced osteoclastogenesis.

The second possible explanation for the normal osteoclastogenesis in *Zip14*‐KO BMMs is that ZIP14 regulates osteoclast‐mediated bone resorption in a non‐cell‐autonomous manner. Among the defects observed in *Zip14*‐KO mice, elevated IL‐6 expression with chronic inflammation is the most likely cause of increased bone resorption. It has been reported that osteoclast formation is triggered by IL‐6 in the presence of soluble IL‐6 receptor (sIL‐6R), and RANKL expression is induced by IL‐6/sIL‐6R via the JAK/STAT signaling pathway 39, 40. *IL6*‐KO mice are protected against ovariectomy‐induced osteoporosis via a mechanism that prevents osteoclast activation 41. Accordingly, anti‐IL‐6R antibody inhibits osteoclast formation in animal models and patients with rheumatoid arthritis 42, 43. Anti‐IL‐6R antibody treatment of *Zip14*‐KO mice would resolve the involvement of elevated IL‐6 levels in the increased bone resorption activity.

Many *in vitro* studies demonstrated that ZIP14 transports other metals (Mn, Fe, and Cd). Among them, Fe has been reported to be involved in osteoclastogenesis 44. Transferrin receptor 1‐mediated Fe uptake promotes osteoclast differentiation and bone resorbing, which are associated with the induction of mitochondrial respiration and the production of reactive oxygen species. Assuming that ZIP14 transports Fe into osteoclasts, Fe levels are predicted to be decreased in *Zip14*‐KO osteoclasts. Therefore, Fe uptake‐mediated promotion of osteoclast differentiation is not likely to be related to the increased bone resorption observed in *Zip14*‐KO mice. Recently, loss‐of‐function mutations in human *ZIP14* were identified in patients with hypermanganesemia and progressive parkinsonism–dystonia 19. In these patients, blood Mn levels are drastically increased, without affecting Zn, Fe, and Cd levels. Consequently, the authors claimed that the major role of ZIP14 is to transport Mn, and ZIP14 dysfunction reduces biliary Mn elimination, causing hypermanganesemia. Strause *et al*. reported that Mn deficiency decreased osteoclast activity in rats fed Mn‐depleted diets 45. It is necessary to examine Mn metabolism in *Zip14*‐KO mice and the relationship between hypermanganesemia and osteoclastogenesis.

In conclusion, the present study illustrates that ZIP14 is involved in the negative regulation of osteoclastogenesis and supports the fact that Zn exerts an inhibitory effect on osteoclast‐mediated bone resorption. Both Zn and other metals transported by ZIP14 may be involved in the ZIP14‐mediated regulation of osteoclastogenesis. Further identification of Zn transporters involved in osteoclastogenesis will help to clarify the role of Zn in bone homeostasis and the pathological mechanism of skeletal abnormalities produced by Zn deficiency.

## Author contributions

SS, MT, and MS performed the main experiments and analyzed the data. SH and TF generated *Zip14*‐KO mice. MT prepared cDNA from RANKL‐treated BMMs and advised about i*n vitro* assay for osteoclastogenesis. TF planned the experiments, analyzed data, and wrote the manuscript.
